# A Multifactorial Case of Stevens-Johnson Syndrome With Atypical Features

**DOI:** 10.7759/cureus.49289

**Published:** 2023-11-23

**Authors:** Jennifer M Paterno, Zach Breslow, Mufit A Mamo, Edgar Mercado

**Affiliations:** 1 Family Medicine, Hospital Corporation of America (HCA) Florida St. Petersburg Hospital, St. Petersburg, USA; 2 Internal Medicine, Nova Southeastern University Dr. Kiran C. Patel College of Osteopathic Medicine, Clearwater, USA; 3 Critical Care, Hospital Corporation of America (HCA) Florida St. Petersburg Hospital, St. Petersburg, USA

**Keywords:** stevens-johnson syndrome (sjs), hypercoagulability, vancomycin infusion, toxic epidermal necrolysis (ten), nikolsky sign

## Abstract

Stevens-Johnson syndrome (SJS) is a severe mucocutaneous reaction that has a broad spectrum of causes and risk factors that include medications and other infectious causes such as *Mycoplasma*. In this case report, a patient with multiple comorbidities that confounded the presentation of Stevens-Johnson syndrome is observed. The patient was a 29-year-old female with a past medical history of recurrent cerebrovascular accident (CVA) who presented for an evaluation of chest pain. After empiric vancomycin was started for suspicion of endocarditis, our patient developed altered mental status, mucositis, and a painful erythematous erosion on her chest concerning for vasculitis, but after treatment and pathological review, it was found to be Stevens-Johnson syndrome. It is important to not forget the wide variety of risk factors for developing Stevens-Johnson syndrome and some of its unique associated presenting symptoms.

## Introduction

Stevens-Johnson syndrome (SJS) and toxic epidermal necrolysis (TEN) are severe mucocutaneous reactions characterized by extensive necrosis and sloughing of the epidermis. They are defined on a spectrum, and clinical diagnoses are distinguished by the percentage of the body surface area (BSA) affected: SJS, <10%; SJS/TEN overlap, 10%-30%; and TEN, >30% [1]. A true diagnosis can be achieved by confirmatory histopathology that shows extensive necrosis and subepidermal separation with negative immunofluorescence [1].

Commonly, 4-28 days after starting an offending agent, patients will develop a prodrome of fever, malaise, and upper respiratory symptoms. Hours to days later, cutaneous manifestations (painful, erythematous macules or atypical targetoid lesions) develop on the face and trunk before spreading to the extremities. These lesions will have a positive Nikolsky sign and skin erosion with gentle pressure [2]. Oral ulceration and mucositis are universal in all cases. A majority of cases have ocular lesions as well; however, other mucosal sites are also commonly involved [3]. Practitioners who are less experienced with SJS/TEN commonly are under the impression that SJS/TEN is a cutaneous disease, but there are numerous systemic complications that can arise, such as acute respiratory distress syndrome, gastrointestinal (GI) necrosis, acute tubular necrosis, hyperglycemia, and even the development of diabetes [4].

In this case report, we describe a patient with an atypical presentation of SJS who had multiple risk factors for developing this condition. We present this case to increase awareness by showing the insidious onset that SJS can have, a true example of multiple risk factors, and the crucial need to keep this rare disease in the differential diagnosis.

## Case presentation

The patient is a 29-year-old female with a medical history significant for uncontrolled insulin-dependent type 2 diabetes mellitus, multiple left parietal/frontal lobe embolic cardiovascular accident (CVA), and elevated factor 8 levels who presented for chest pain. She initially reported that this pain began two days prior to presentation to the emergency department, was sharp in nature, and was diffusely located across her right and left chest wall. She stated that nothing made the pain better or worse, and it was not associated with deep breaths. The patient also reported that she had a persistent rash diffusely located on her bilateral groin, bilateral upper extremities, and chest for almost a year. She stated that this rash began about the same time when she was hospitalized for her initial CVA. Of note, the patient's mother died early in her 30s secondary to a stroke.

In the emergency department, the patient was found to be hypertensive as well as hyperglycemic with a blood sugar of 326. Cardiac troponin was within normal limits, as well as the subsequent follow-up. The patient's electrocardiogram indicated normal sinus rhythm without any T-wave or ST abnormalities. A computed tomography (CT) angiogram of the chest was significant for diffuse bilateral pulmonary nodules; differentials included metastatic disease versus infectious process.

**Figure 1:**
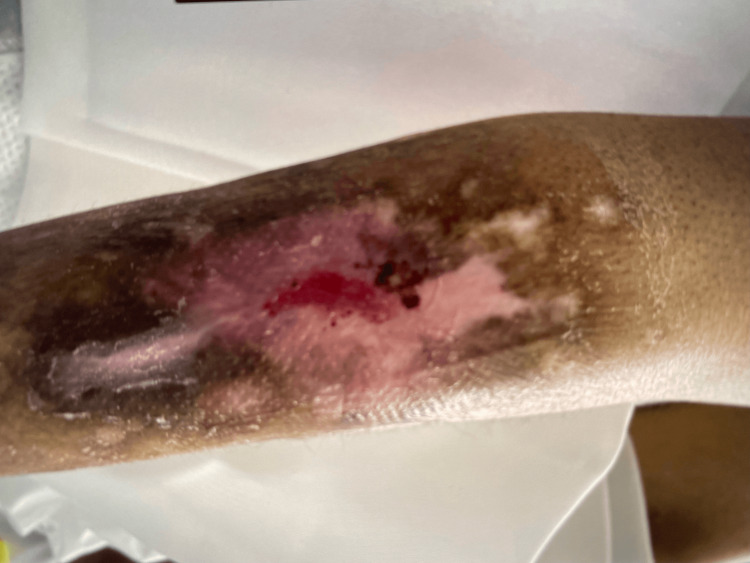
Upper extremity cutaneous skin lesion This image demonstrates findings consistent with late stages of toxic epidermal necrolysis.

Urinalysis was also significant for many white blood cells, many bacteria, and leukocyte esterase. On physical examination, the patient was noted to have chronic bilateral anterior lower extremity ulcers, an erythematous rash located on the groin, and what appeared to be lesions of various healing stages on bilateral upper extremities as seen in Figure 1. Blood cultures were then obtained for suspicion of sepsis secondary to urinary tract infection versus septic pulmonary nodules. She was started on vancomycin and ceftriaxone and admitted to the medicine floor with telemetry monitoring.

On hospital day 1, a transthoracic echocardiogram (TTE) was obtained for suspicion of endocarditis. The study showed a possible tricuspid mass/vegetation; however, this is a limited study. Vancomycin and ceftriaxone were continued, and a transesophageal echocardiogram (TEE) was obtained. Cardiology and Infectious Disease were consulted at this time. On hospital day 3, the TEE showed normal systolic function and was negative for any evidence of vegetation on all four valves.

**Figure 2:**
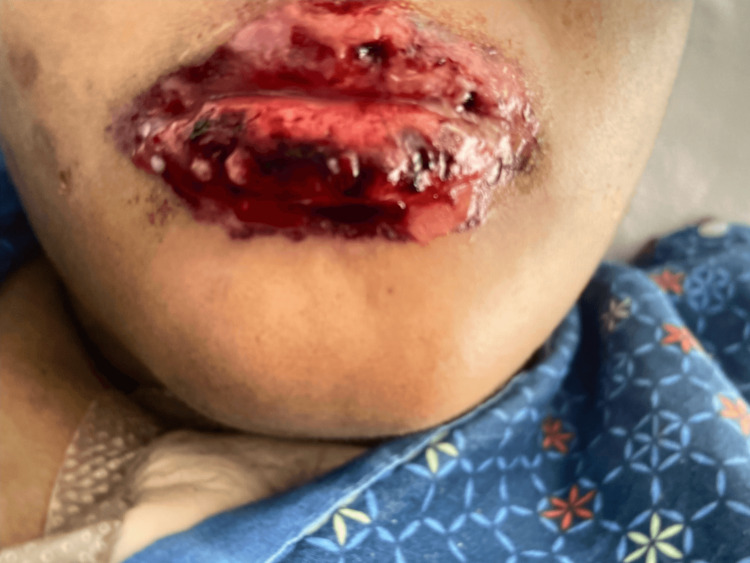
Labium superius and labium inferius oris This image displays hemorrhagic erosions of mucous membranes, which is a key feature of Stevens-Johnson syndrome.

On hospital day 5, the patient had a change in her mental status from baseline and was noted to have mucositis of her lips as seen in Figure 2. A magnetic resonance imaging (MRI) of the brain was significant for acute ischemia involving the left middle cerebral artery (MCA) with involvement of the posterior left frontal lobe, left temporal lobe, and left parieto-occipital lobe. Punctate ischemia involving the left basal ganglia was also noted. Old infarcts involving the left frontal and parietal lobes were noted on MRI. At this time, vancomycin was discontinued after ruling out endocarditis via TEE.

On hospital day 7, workups for lupus, vasculitis, and coagulopathy were negative. Mucositis progressively worsened, and on hospital day 9, she was upgraded to the intensive care unit. A punch biopsy of a new active left upper chest lesion was obtained and sent for histopathology. Treatment with high-dose steroids (methylprednisolone 1 gram daily) was started for suspected Moyamoya disease versus seronegative vasculitis. The patient showed significant improvement with high-dose steroids and began to return to her mental baseline. Histopathology was significant for epidermal changes showing dyskeratotic keratinocytes and subepidermal separation, consistent with SJS. The patient was subsequently discharged to inpatient rehabilitation for aggressive physical therapy following acute CVA.

## Discussion

The exact cause of SJS/TEN is unknown, but approximately 75% of cases are medication-induced [5]. It is believed that medications and/or their metabolites form a hapten and are taken up by antigen-presenting cells causing CD8+/CD4+ T cell activation [5]. Individuals are stratified on their risk based on genetic and systemic comorbidities. SJS/TEN more commonly affects older adults [5]. Specific human leukocyte antigens (HLAs) put some populations at an increased risk of SJS/TEN. For example, HLA-B*58:01 is associated with an increased risk on allopurinol in Taiwanese, Japanese, Korean, Thai, and European populations [2]. The most commonly implicated drugs include antiepileptics (phenytoin, lamotrigine, carbamazepine, and phenobarbital), sulfa antibacterial (sulfamethoxazole), allopurinol, nonsteroidal anti-inflammatory drugs (NSAIDs) of the oxicam type (piroxicam), and nevirapine (non-nucleotide reverse transcriptase inhibitor) [5].

Less commonly, antibiotics are another group of medications that can cause SJS [5]. Vancomycin, which covers methicillin-resistant *Staphylococcus aureus*, is the most widely prescribed antibiotic in United States hospitals [6]. A cross-sectional study of vancomycin drug allergy labels in electronic health records at Johns Hopkins Health System Corporation or Mass General Brigham between January 2017 and December 2019 found a total of 11,825 vancomycin hypersensitivity reactions (HSRs) out of 4,490,618 cases. Of those HSRs, there were 34 cases of vancomycin-induced SJS (0.3%) and two cases of vancomycin-induced TEN (0.02%). Out of this population, there were a total of 136 severe cutaneous adverse reactions (SCARs) (including drug reaction with eosinophilia and systemic symptoms (DRESS), SJS/TEN, and acute generalized exanthematous pustulosis (AGEP)). The most common vancomycin-induced SCAR was DRESS with 82 cases, followed by SJS/TEN with 34 cases [6].

Ni et al. conducted post-marketing surveillance of SJS secondary to vancomycin and linezolid from the FDA's Adverse Event Reporting System (FAERS) between January 2004 and July 2021 [7]. There were a total of 276 vancomycin-induced SJS in this time period compared to 63 cases from linezolid. Of the vancomycin population, the most common age range affected was between 45 and 64 years old at 31.5%, while 14.9% of patients were between the ages of 18 and 44 years old. The distribution based on gender was 49.3% and 43.8% in males and females, respectively. The time of onset of vancomycin-induced SJS was 12 days compared to linezolid of five days [7].

Infectious Stevens-Johnson syndrome

A smaller percentage of SJS is thought to be caused by pathogens, including *Mycoplasma pneumoniae* and viruses [5]. *Mycoplasma pneumoniae* typically causes asymptomatic to mild respiratory tract infections. However, around 25% of cases have extrapulmonary manifestations, including stroke, carditis, erythema multiforme (EM), SJS, and *Mycoplasma pneumoniae*-associated mucositis (MPAM) (also known as atypical SJS) [8]. These manifestations can occur in the absence of pulmonary symptoms and are more common in children and adolescents [8]. Chiang et al. found that subjects with a history of *M. pneumoniae* infection had higher rates of chronic kidney disease (1.9% versus 3%; p<0.001), coronary artery disease (4.5% versus 5.6%; p<0.001), heart failure (0.9% versus 1.8%; p<0.001), and ischemic stroke (1.1% versus 0.72%; p=0.01) [9]. The pathology is not completely understood, but some proposed mechanisms include postinfectious autoimmune phenomenon, vascular occlusions secondary to vasculitis or hypercoagulable state, and adenosine diphosphate ribosylating toxin [8].

In 2014, the term *Mycoplasma*-induced rash and mucositis (MIRM) was introduced as an entity separate from SJS/TEN. MIRM typically affects younger patients (8-11 years old) in the winter months and follows prodromal symptoms a week prior. The skin involvement in MIRM typically affects only the mucosa; however, in 47% of cases, there is acral skin involvement [10]. Less commonly, 23% of cutaneous lesions involved the chest [10]. Reported cases of MIRM have identified different cutaneous lesions, including vesiculobullous, targetoid, papular, macular, and morbilliform [10]. Diagnosis is made with serological testing with cold agglutinins, cultures, and polymerase chain reaction (PCR) [10]. It is worth noting that our patient worked as a chef at a local rehabilitation site.

A rare complication of *Mycoplasma pneumoniae* infections is ischemic stroke, and as of 2022, there were 28 reported cases, of which nine occurred in adults. The typical presenting symptom in these nine cases was pneumonia or an upper respiratory infection. Of these patients, seven were females. Most of these cases were in their late 20s-30s. They additionally were all diagnosed with *Mycoplasma* by cold agglutinin or complement fixation [8].

Around 5%-20% of cases of SJS are idiopathic. Some rare causes of SJS include vaccination, bone marrow transplants (although this is difficult to distinguish from graft versus host disease), radiation therapy, irritable bowel disease, IV contrast administration, and toxic chemical exposure [4].

In many cases of SJS, the treatment involves discontinuing the offending agent. In this case, vancomycin had been discontinued after ruling out bacterial endocarditis before there was a suspicion for an adverse drug reaction. Treating with steroids for suspected vasculitis is the same treatment as SJS, which resulted in marked improvement in the patient's condition.

## Conclusions

Prompt recognition of SJS is key to treatment as early intervention can spare patients from numerous complications. In the case of this patient, it may be difficult to recognize the cause immediately. With the patient's history of recurrent CVAs, the new altered mental status, and mucosal erosions, vasculitis was also high on the differential. It is unclear if there is an association with SJS and her history of recurrent CVA. While SJS can cause hypercoagulability, it typically presents with a deep vein thrombosis (DVT) or pulmonary embolism (PE) rather than an ischemic stroke. Despite the patient's numerous risk factors and questionable autoimmune disease, it is believed that this patient's SJS was induced by vancomycin as the mucosal changes and altered mental status started five days after starting vancomycin and resolved with treatment after removing the offending agent and IV steroids.
